# Syk Plays a Critical Role in the Expression and Activation of IRAK1 in LPS-Treated Macrophages

**DOI:** 10.1155/2017/1506248

**Published:** 2017-06-07

**Authors:** Jae Gwang Park, Young-Jin Son, Byong Chul Yoo, Woo Seok Yang, Ji Hye Kim, Jong-Hoon Kim, Jae Youl Cho

**Affiliations:** ^1^ Department of Genetic Engineering, Sungkyunkwan University, Suwon 16419, Republic of Korea; ^**2**^ Department of Pharmacy, Sunchon National University, Suncheon 57922, Republic of Korea; ^3^Research Institute and Hospital, National Cancer Center, Goyang 10408, Republic of Korea; ^**4**^ Department of Physiology, College of Veterinary Medicine, Chonbuk National University, Iksan 54596, Republic of Korea

## Abstract

To address how interleukin-1 receptor-associated kinase 1 (IRAK1) is controlled by other enzymes activated by toll-like receptor (TLR) 4, we investigated the possibility that spleen tyrosine kinase (Syk), a protein tyrosine kinase that is activated at an earlier stage during TLR4 activation, plays a central role in regulating the functional activation of IRAK1. Indeed, we found that overexpression of myeloid differentiation primary response gene 88 (MyD88), an adaptor molecule that drives TLR signaling, induced IRAK1 expression and that piceatannol, a Syk inhibitor, successfully suppressed the MyD88-dependent upregulation of IRAK1 under LPS treatment conditions. Interestingly, in Syk-knockout RAW264.7 cells, IRAK1 activity was almost completely blocked after LPS treatment, while providing a Syk-recovery gene to the knockout cells successfully restored IRAK1 expression. According to our measurements of IRAK1 mRNA levels, the transcriptional upregulation of IRAK1 was induced by LPS treatment between 4 and 60 min, and this can be suppressed in Syk knockout cells, providing an effect similar that that seen under piceatannol treatment. The overexpression of Syk reverses this effect and leads to a significantly higher IRAK1 mRNA level. Collectively, our results strongly suggest that Syk plays a critical role in regulating both the activity and transcriptional level of IRAK1.

## 1. Introduction

Inflammation, which commonly accompanies redness, pain, swelling, and heat, is one of the most important barriers in innate immunity. This process enables the body to recognize regions that are infected or damaged and therefore in need of repair. The inflammatory response is controlled by several immune cells, such as macrophages, dendritic cells, neutrophils, and eosinophils [1–3]. These cells express several receptors, including the toll-like receptors (TLR), which recognize exogenous pathogens or infected materials and then promote the activation of these receptors, thereby triggering a variety of cellular responses, including the expression and activation of inflammation regulatory genes such as cytokines, adhesion molecules, costimulatory molecules, and chemokines [4–7]. The transcription and translation of these genes rely on the activation of additional transcription factors as well as their upstream signaling cascades [8].

Of these upstream signaling proteins, interleukin-1 receptor-associated kinase 1 (IRAK1) has been reported to be a pivotal regulator of the TLR-derived signaling pathways. When TLRs are stimulated by their counter ligands, such as lipopolysaccharide (LPS) in the case of TLR4, myeloid differentiation primary response gene 88 (MyD88) and TIR-domain-containing adaptor-inducing interferon-*β* (TRIF) participate in delivering the inflammation activation signals within the cell [9]. It is known that IRAK1 activates TNF receptor-associated factors (TRAF) and transforming growth factor beta-activated kinase 1 (TAK1), which simultaneously induce both nuclear factor- (NF-) *κ*B and activator protein- (AP-) 1 activation signals [10]. Prior work has shown that the pharmacological inhibition of IRAK1 strongly inhibits inflammatory responses by suppressing both NF-*κ*B and AP-1, demonstrating that IRAK1 holds critical functional significance in inflammatory signaling and responses.

Much previous work has suggested that IRAK1 provides an excellent target enzyme for anti-inflammatory drugs; yet, the ways in which this enzyme is regulated by other signaling proteins at the transcriptional, translational, and posttranslational levels remain largely unknown. For this study, we employed the CRISPR/Cas9 system to generate Syk knockout cells and we quantified both the protein and mRNA levels of IRAK1. We report evidence that spleen tyrosine kinase (Syk), a representative protein tyrosine kinase, acts as a key regulator in IRAK1 expression and activation during the TLR4 activation process triggered by LPS treatment in macrophages.

## 2. Materials and Methods

### 2.1. Materials

Polyethylenimine (PEI), 3-(4,5-dimethylthiazol-2-yl)-2,5-diphenyltetrazolium bromide (a tetrazole) (MTT), sodium dodecyl sulfate (SDS), dimethyl sulfoxide (DMSO), and lipopolysaccharide (LPS, *E. coli* 0111:B4) were purchased from Sigma Chemical Company (St. Louis, MO, USA). Piceatannol was obtained from Calbiochem (La Jolla, CA, USA). Fetal bovine serum (FBS), penicillin, streptomycin, TRIzol Reagent, and RPMI1640 were obtained from GIBCO (Grand Island, NY, USA). RAW264.7 cells, a murine macrophage-like cell line, and HEK293 cells, a human embryonic kidney cell line, were purchased from ATCC (Rockville, MD, USA). Lipofectamine 2000 was purchased from Thermo Fisher (USA). All other chemicals used in this study were of analytical grade and purchased from Sigma Chemical Company. Phospho-specific or total-protein antibodies to IRAK1, IRAK4, MyD88, Syk, Myc, TAK1, and *β*-actin were purchased from Cell Signaling Technology (Beverly, MA, USA).

### 2.2. Expression Vectors

Myc-Syk, Flag-IRAK1, and Flag-MyD88 were used as previously reported [11, 12]. Luciferase plasmids containing binding sites for NF-*κ*B or AP-1, as well as an adaptor molecule (MyD88) for TLR signaling, were used as previously reported [13]. Plasmid sequences were checked by automated DNA sequencing.

### 2.3. Cell Culture and Drug Preparation

RAW264.7 cells, a murine macrophage cell line, and HEK293 cells were maintained in RPMI1640 media supplemented with 100 U/ml of penicillin, 100 *μ*g/ml of streptomycin, and 10% FBS. The cells were grown at 37°C and 5% CO_2_ in humidified air. A stock solution of piceatannol was prepared in DMSO.

### 2.4. Preparation of Syk Knockout Cell Line

Syk knockout cells were established using the CRISPR/Cas9 system on RAW264.7 cells. The sgRNAs library sequences targeting Syk were evaluated using the CRISPR Design tool (cripr.mit.edu). One of the Syk-targeted sgRNA library sequences (F-CACCGACCCAGGTAGTTGCGGCTC, R-AAACGAGCCGCAACTACCTGGGTC) was chosen and cloned into the PX459 pSpCas9 vector (Addgene plasmid ID #48139). Plasmids were transfected into RAW264.7 cells with Lipofectamine 2000 according to the manufacturer's protocol. After 48 h, cells were treated with puromycin (1–3 *μ*g/ml) for 72 hours. After 5 days of culture, the cells selected by puromycin were diluted and plated individually in a 96-well plate and cultured for 2-3 weeks in media containing puromycin (1 *μ*g/ml). Then, 25 single clones were harvested and evaluated by Western blot for Syk protein expression. Cells that demonstrated no Syk protein expression were used for subsequent experiments.

### 2.5. mRNA Analysis Using Polymerase Chain Reaction

In order to test cytokine mRNA expression levels, total RNA was isolated from LPS-activated macrophage-like RAW264.7 cells using TRIzol Reagent, according to the manufacturer's instructions. Total RNA was stored at −70°C until use. mRNA quantification was carried out by real-time polymerase chain reaction (real-time PCR) with SYBR Premix Ex Taq according to the manufacturer's instructions (Takara, Shiga, Japan) using a real-time thermal cycler (Bio-Rad, Hercules, CA, USA), as previously reported [14, 15]. All of the primers were synthesized by Bioneer (Bioneer, Daejeon, Korea) and are listed in Table 1.

### 2.6. Preparation of Whole Cell Lysates and Nuclear Fractions for Immunoblotting

RAW264.7 or HEK293 cells (5 × 10^6^ cells/ml) were washed 3 times in cold PBS containing 1 mM sodium orthovanadate and lysed in lysis buffer (20 mM Tris-HCl, pH 7.4, 2 mM EDTA, 2 mM ethyleneglycotetraacetic acid, 50 mM *β*-glycerophosphate, 1 mM sodium orthovanadate, 1 mM dithiothreitol, 1% Triton X-100, 10% glycerol, 10 *μ*g/ml aprotinin, 10 *μ*g/ml pepstatin, 1 mM benzimide, and 2 mM PMSF) for 30 min with rotation, at 4°C as reported previously [16]. All lysates were clarified by centrifugation at 16,000 ×g for 10 min at 4°C and stored at −20°C until needed.

Whole cell lysates were subsequently analyzed by immunoblotting. Proteins were separated on 10% SDS-polyacrylamide gels and transferred by electroblotting to polyvinylidene difluoride (PVDF) membranes. Membranes were blocked for 60 min in Tris-buffered saline containing 3% FBS, 20 mM NaF, 2 mM EDTA, and 0.2% Tween 20 at room temperature. The membranes were incubated for 60 min with specific primary antibodies at 4°C, washed 3 times with the same buffer, and incubated for an additional 60 min with HRP-conjugated secondary antibodies. The total and phosphorylated levels of IRAK1, IRAK4, MyD88, Syk, Myc, TAK1, and *β*-actin were visualized using an ECL system (Amersham, Little Chalfont, Buckinghamshire, UK), as previously reported [17].

### 2.7. DNA Transfection and Luciferase Reporter Gene Activity Assay

Overexpression experiments were performed with HEK293 cells (5 × 10^6^ cells/ml) or RAW264.7-Syk^−/−^ cells by transfection with MyD88, Flag-IRAK1, or Syk-WT using the PEI method in 12-well plates, as previously reported [18, 19]. Cells were used for experiments 24 h after transfection. Piceatannol was added to cells 12 h before termination. For reporter gene assays, HEK293 cells (1 × 10^6^ cells/ml) were transfected with 1 *μ*g of plasmids containing constitutively expressed NF-*κ*B-Luc and *β*-galactosidase genes using the PEI method in 12-well plates, as previously reported [18, 19]. Luciferase assays were performed using the Luciferase Assay System (Promega, Madison, WI, USA), as previously reported [20].

### 2.8. Statistical Analyses

All data are presented as mean ± SD based on a minimum of three independent replicate experiments. For statistical comparisons, results were analyzed using either ANOVA/Scheffe's post hoc test or the Kruskal-Wallis/Mann–Whitney test. A *P* value < 0.05 was considered to indicate a statistically significant difference. All statistical tests were carried out using SPSS (SPSS Inc., Chicago, IL, USA). Similar experimental data were observed using an additional independent set of in vitro experiments that were conducted using the same numbers of samples or mice.

## 3. Results

### 3.1. Piceatannol Suppresses MyD88-Dependent IRAK1 Activation

**Figure 1:**
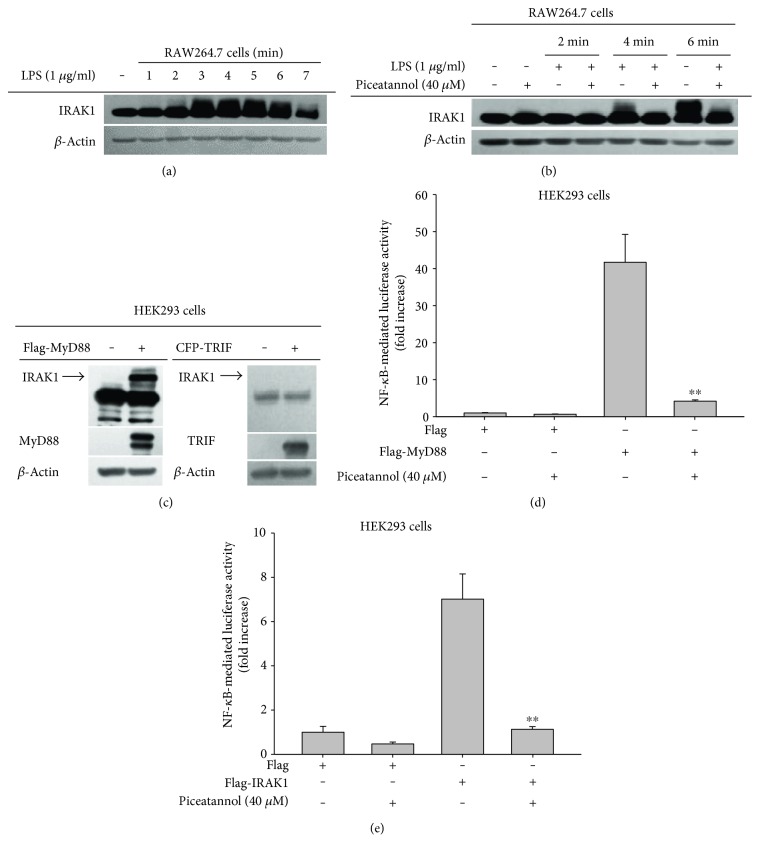
Piceatannol impacts IRAK1 activation during LPS treatment in RAW264.7 cells. (a, b, and c) Protein levels of IRAK1, IRAK4, MyD88, and *β*-actin as determined by lysate immunoblotting prepared from LPS- (1 *μ*g/ml) treated RAW264.7 cells or MyD88-overexpressed HEK293 cells. (d and e) HEK293 cells cotransfected with NF-*κ*B-Luc (1 *μ*g/ml) and *β*-gal (as a transfection control) plasmid constructs were treated with piceatannol (40 *μ*M) in the presence or absence of an adaptor molecule (MyD88) or IRAK1 for 12 h. Luciferase activity was determined via luminometry. All data are expressed as the mean ± SD of three independent experimental replicates. ^∗∗^*p* < 0.01 compared to the control group.

To determine whether Syk regulates IRAK1 during TLR4 activation in macrophages, we first quantified IRAK1 expression in LPS-treated RAW264.7 cells. IRAK1 was found to increase its expression level between 3 and 6 min after LPS treatment (Figure 1(a)). After 7 min, IRAK1 reached its basal expression level (Figure 1(a)). Next, we assessed whether treatment with piceatannol (40 *μ*M), a Syk inhibitor, suppressed IRAK1 activation after LPS treatment. Interestingly, we found that it strongly suppressed the activation of IRAK1 at 4 and 6 min after LPS treatment (Figure 1(b)). In order to assess whether this critical adaptor molecule regulates the activation and expression of IRAK1, we next quantified the expression level of IRAK1 using immunoblotting analysis under overexpression conditions induced by transfection with MyD88 or TRIF in HEK293 cells using methods previously reported [21]. The overexpression of MyD88 without overexpression of TRIF strongly increased the protein expression level of IRAK1 (Figure 1(c)). Next, we confirmed whether MyD88-induced NF-*κ*B activation was also regulated by piceatannol. We found that this inhibitor strongly suppressed NF-*κ*B-mediated luciferase activity that is typically induced by MyD88 (Figure 1(d)). Finally, we tested whether IRAK1 activity can be blocked by piceatannol using a luciferase assay in which IRAK1 overexpression increased NF-*κ*B activity, which in turn would lead to increased luciferase activity. We found that piceatannol strongly reduced the luciferase activity, thereby confirming its role in the suppression of IRAK1 activity (Figure 1(e)).

### 3.2. Syk Regulates IRAK1 Activation in LPS-Treated RAW264.7 Cells

**Figure 2:**
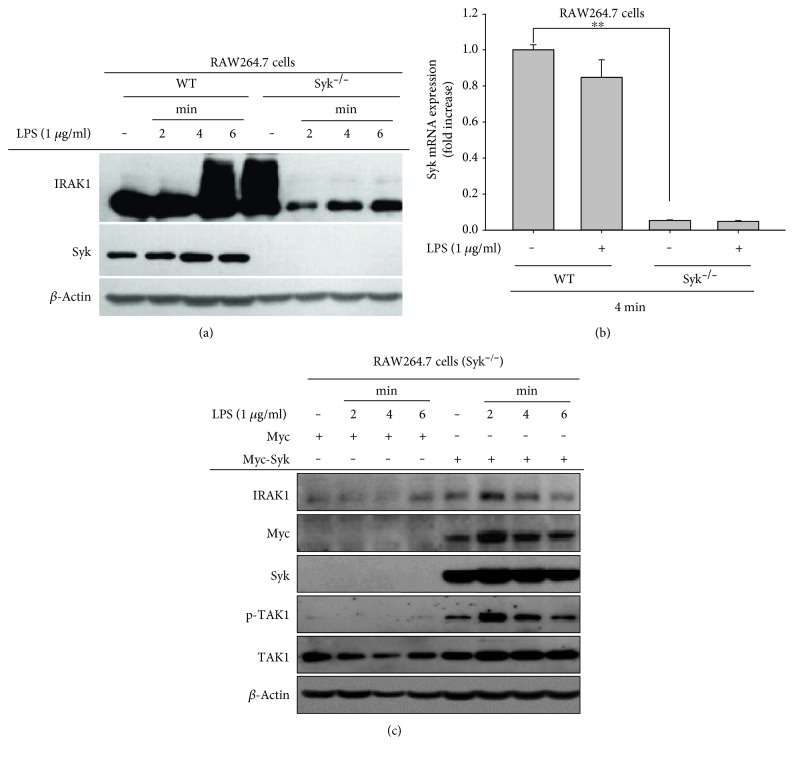
Syk impacts the regulation of IRAK1 activation in LPS-treated RAW264.7 cells. (a and c) Total or phospho-protein levels of IRAK1, Syk, Myc, TAK1, and *β*-actin as determined by lysate immunoblotting prepared from LPS- (1 *μ*g/ml) treated RAW264.7-WT, RAW264.7-Syk^−/−^ cells, or RAW264.7-Syk^−/−^ cells transfected for 24 h with Myc-tagged Syk (5 × 10^6^ cells/ml). (b) Syk mRNA levels from RAW264.7-WT or RAW264.7-Syk^−/−^ cells treated with LPS (1 *μ*g/ml) as determined using real-time PCR. All data are expressed as the mean ± SD of three independent replicate experiments.

We next sought to characterize the potential MyD88-mediated connection between Syk and IRAK1 by measuring the effect of Syk on IRAK1 regulation after LPS treatment. To this end, we prepared Syk knockout (Syk^−/−^) RAW264.7 cells, which we confirmed to exhibit low or null Syk mRNA and protein levels (Figure 1(a) left and right panels). We further measured the protein level of IRAK1 in both wild-type (WT) and Syk^−/−^ cells after LPS treatment. IRAK1 was strongly induced at 4 and 6 min after LPS treatment in wild-type (WT) RAW264.7 cells; however, there was no induction of IRAK1 at any point after LPS-treatment in Syk^−/−^ cells (Figure 1(a)). We subsequently transfected an expression plasmid containing a functional Syk gene into Syk^−/−^ cells, and it showed strikingly upregulated IRAK1 expression between 2 and 4 min (Figure 2(c)), demonstrating a tight regulatory effect of Syk on IRAK1.

### 3.3. Syk Regulates IRAK1 Transcription in LPS-Treated RAW264.7 Cells

**Figure 3:**
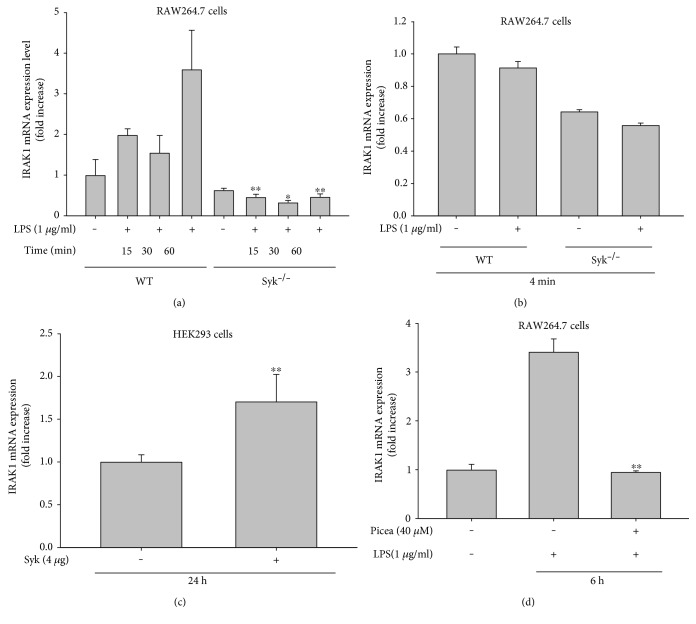
Syk impacts the transcriptional regulation of IRAK1 in LPS-treated RAW264.7 cells. (a and b) IRAK1 mRNA levels from RAW264.7-WT or RAW264.7-Syk^−/−^ cells with or without LPS (1 *μ*g/ml) treatment, as determined by real-time PCR. (c) IRAK1 mRNA levels from HEK293 cells transfected with Syk, as determined by real-time PCR. All data are expressed as the mean ± SD of three independent replicate experiments. ^∗^*p* < 0.05 and ^∗∗^*p* < 0.01 compared to the control group (LPS alone) or normal group.

To understand how Syk regulates IRAK1 activity, we next quantified IRAK1 mRNA levels in Syk^−/−^ cells. As expected, the IRAK1 mRNA expression remained at a basal level after LPS treatment, whereas LPS caused 2- or 3-fold increased expression in WT RAW264.7 cells between 15 and 60 min after treatment (Figure 3(a)). IRAK1 mRNA levels were not affected by LPS treatment after 4 min in either WT or Syk^−/−^ cells (Figure 3(b)). Syk overexpression, however, significantly upregulated IRAK1 mRNA level by up to 2-fold (Figure 3(c)), supporting the possibility that Syk activity plays a critical role in promoting IRAK1 gene expression. Lastly, we assessed whether piceatannol successfully suppresses IRAK1 mRNA expression after LPS treatment. We found that piceatannol (40 *μ*M) significantly downregulated IRAK1 transcription after LPS treatment in RAW264.7 cells (Figure 3(d)).

## 4. Discussion

IRAK1 is a serine/threonine kinase that is normally activated by TLR4/MyD88 interaction in macrophages and dendritic cells [22]. Prior work has implicated IRAK1 in regulating NF-*κ*B activation signaling, which in turn regulates the expression of various cytokines as well as genes that generate reactive oxygen species [23]. It is known that IRAK1 interacts with TRAF6, promoting additional downstream signaling events, including the activation of IKK [24]. When IRAK1 is suppressed by small molecule treatment, it has been shown to block the TLR4-mediated inflammatory response, as well as DSS- or LPS-induced colitis and septic shock [25, 26]. As such, we consider IRAK1 to be a particularly promising target for treating or curing inflammatory diseases [22]. Despite the potential pharmacological value of IRAK1 in inflammatory diseases, the mechanisms that regulate its expression and activity remain largely unknown.

With this work, we strongly suggest that Syk is a critical factor regulating the activation and expression of IRAK1. We showed that IRAK1 activation was significantly increased between 3 and 6 min after LPS treatment, since IRAK1 activation was accompanied by ubiquitination, and the protein band corresponding to high-molecular weight IRAK1 significantly increased during that time period (Figure 1(a)). Interestingly, we found that, under Syk knockout conditions, the high-molecular-weight IRAK1 product was completely blocked (Figure 2(a)), whereas transfection of constitutively expressed Syk into Syk^−/−^ cells restored the level of high-molecular-weight IRAK1 (Figure 2(c)), implying that Syk is an important enzyme regulating the activation of IRAK1. Additionally, we found a significant reduction in IRAK1 gene expression in Syk ^−/−^ cells (Figure 3(a)), indicating that Syk also plays a central role in regulating the mRNA and protein levels of IRAK1. We identified similar regulatory effects at the transcriptional and posttranslational levels by pharmacological interference after treating cells with piceatannol, a Syk inhibitor (Figures 1(b) and 3(d)). In fact, the important role of Syk in inflammatory responses was also reported previously [1]. Thus, we have found that Syk acts as a critical enzyme to activate early NF-*κ*B pathway in macrophages under TLR4 stimulation conditions [27]. Inhibition of Syk was also linked to suppression of the production of inflammatory mediators [28, 29]. In addition, it was reported that TLR4 is tightly associated with Syk [30, 31]. Therefore, these results strongly suggest that TLR-associated Syk might functionally associate with IRAK1 controlled by MyD88 and TLR4.

The expression of IRAK1 was found to be MyD88-dependent. In accordance with previous results [32, 33], we showed that the overexpression of MyD88, but not TRIF, triggered IRAK1 upregulation (Figure 1(c)), indicating that MyD88 is required for IRAK1 expression and activation. Additionally, we found that the activation of NF-*κ*B by IRAK1 (Figure 1(e)) also occurred under MyD88 overexpression conditions (Figure 1(d)), implying that IRAK1 is one of the critical downstream components driving MyD88-mediated NF-*κ*B activation. Since piceatannol blocked IRAK1- and MyD88-induced NF-*κ*B activation (Figures 1(d) and 1(e)), we concluded that Syk might be linked to this NF-*κ*B activation pathway as an upstream regulator. To date, we are aware of no findings that indicate that IRAK1 engages in crosstalk with Syk in inflammatory signaling. Therefore, if we are to understand the interregulatory mode of action between these enzymes and whether they are biochemically associated in the process driving NF-*κ*B activation under MyD88 stimulation, it will need to be examined using immunoprecipitation and immunoblotting analysis.

Recently, we found that several chemical compounds, including anthraquinone-2-carboxlic acid, caffeic acid, and kaempferol, and extracts from medicinal plants such as *Torreya nucifera*, *Myrsine seguinii*, *Rhodomyrtus tomentosa*, *Osbeckia stellata*, and *Polygonum hydropiper* act as dual inhibitors in blocking Syk and IRAK1 activity [34–39]. Previous work from other groups have also shown that IRAK1 inhibitors such as 2-hydroxy-5,6-dihydroisoindolo[1,2-a]isoquinoline-3,8-dione, mangiferin, and corosolic acid and the mixture of the rhizomes of *Anemarrhena asphodeloides* and *Coptidis chinensis* exhibit anti-inflammatory activities when applied to in vivo endotoxemia and colitis animal models [25, 32, 40, 41]. Together, these results strongly suggest that IRAK1 positively regulates numerous inflammatory diseases and therefore has significant potential to be targeted in order to treat a broad range of inflammatory diseases. Supporting this idea, it was also found that IRAK1-deficient mice exhibit markedly lower levels of interleukin- (IL-) 6 and IL-1*β* and suffer from significantly less sepsis-induced mortality [42]. Additionally, it was shown that the deletion of IRAK1 promotes cardiac contractile dysfunction [43]. There is also a possibility that IRAK1 and Syk have a high degree of structural similarity, which could be simultaneously recognized by some dual inhibitors, although there do not appear to be any shared functional domains between them. Whether these dual inhibitors interact pharmacologically with both IRAK1 and Syk using the same structural units in each chemical remains to be tested, in particular by synthesizing analogs of anthraquinone-2-carboxlic acid, caffeic acid, and kaempferol. We acknowledge, however, that this may prove difficult to explore.

**Figure 4:**
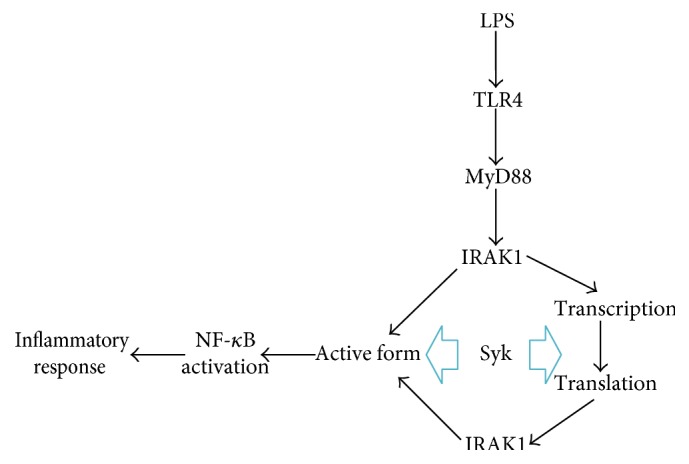
Schematic pathway for Syk regulation of IRAK1 expression and activity.

In summary, we demonstrated that Syk might be a critical enzyme that regulates IRAK1 activity and expression in macrophages stimulated by LPS. We showed that treatment with the Syk inhibitor piceatannol, as well as Syk^−/−^ knockout cells, leads to dramatically reduced level of the IRAK1 active form. Also, we found that IRAK1 mRNA expression was also diminished in both the Syk^−/−^ knockout cells and in cells treated with piceatannol. Our results collectively imply that Syk acts as an important regulator of IRAK1 during inflammatory responses by controlling it at both the transcriptional and posttranslational levels, as summarized in Figure 4. The molecular mechanisms whereby Syk modulates these processes remain to be identified, and further studies focused on such mechanistic details will follow.

## Figures and Tables

**Table 1:** Real-time PCR primers used in this study.

Name	Sequence (5′ to 3′)	
Syk	F	CAGGAACCTGATGGCCTTAT
	R	CCTGAAGGTTCCAGGTCTGT
IRAK1	F	CACTTCTTGTACGAGGTGCCA
	R	GATGGGGAACATCAGGCTTCA
GAPDH	F	CAATGAATACGGCTACAGCAAC
	R	AGGGAGATGCTCAGTGTTGG

## Abbreviations

IRAK1: Interleukin-1 receptor-associated kinase 1
TLR: Toll-like receptors
NF-*κ*B: Nuclear factor-*κ*B
AP-1: Activator protein-1
JNK: c-Jun N-terminal kinase
MTT: 3-(4,5-Dimethylthiazol-2-yl)-2,5-diphenyltetrazolium bromide (a tetrazole)
DTT: Dithiothreitol
PI3K: Phosphoinositide 3-kinase
LPS: Lipopolysaccharide
PCR: Reverse transcriptase polymerase chain reaction.
